# Technological properties and composition of volatile compounds in winter wheat malts grown with addition of seed meals into soil

**DOI:** 10.1038/s41598-023-27803-0

**Published:** 2023-01-12

**Authors:** Alan Gasiński, Elżbieta Pytlarz, Oliwia Hamkało, Joanna Kawa-Rygielska

**Affiliations:** 1 Department of Fermentation and Cereals Technology, Wrocław University of Environmental and Life Sciences, Chełmońskiego 37, 51-630 Wrocław, Poland; 2 Institute of Agroecology and Plant Production, Wrocław University of Environmental and Life Sciences, Grunwaldzki Square 24A, 50-363 Wrocław, Poland

**Keywords:** Nutrition, Plant domestication, Plant physiology

## Abstract

Wheat malts are necessary to produce wheat beers. In this study, wheat was grown with addition of seed meals into the soil, to determine whether such agricultural practice could improve the quality of the grain and, therefore, improve the quality of wheat malt produced from these grains. It was determined, that malt produced from the grains of the winter wheat cultivated with the use of seed meals is characterised with improved technological properties, such as saccharification time, filtration time and extract content and some of the seed meals had a positive impact on the content of various volatile compounds present in the produced malts.

## Introduction

Malting is a procedure typically used to produce malt from the barley (*Hordeum vulgare* L.), which is mainly used by the brewing industry, to produce wort. Wort then can be fermented to acquire the final product: beer. The second most important grain, which is malted and used in the brewing industry for the production of alcoholic beverages is wheat (*Triticum aestivum* L.). There are various beer types (called ‘beer styles’), such as hefeweizen, wheatwine or berliner weisse in which part of the mashed grist consists of malted wheat^1^. The goal of malting is production and activation of various enzymes, such as amylases, proteases and beta-glucanases, which are then used in the mashing process to hydrolyse starch, proteins and a variety of different components present in the malted grains. Malting also modifies the physical structure of the grain and changes grain colour, taste and aroma^2^. The main processes during the malting are: steeping, in which grain moisture is increased; germination, during which majority of metabolic changes, such as enzyme production occur; kilning (drying) during which metabolism of the budding grain is topped and the storage stability of the material is increased^3^. Quality of the malts mainly depends on the quality of raw material used for the process of malting^4^. In recent years, the main idea in agriculture has been to ensure the sustainable development and preservation of the agroecosystem biodiversity. The increase in the problem of weed resistance to herbicides^5^, the withdrawal of active substances and the limitation of their use are in accordance with both pro-ecological production and integrated plant management promoted. It is an expected practice in plant cultivation. It includes, among other things, the use of the allelopathic potential of crops in the form of living mulches, catch crops or seed meals, which can reduce weed infestation (including herbicide-resistant biotypes) and improve crop growth^6,7^. The allelochemicals, which can be found in various parts of plants, such as stems, roots, leaves or seeds may have an influence on variety of metabolic processes, such as uptake of ions, respiration, function of enzymes, photosynthesis as well as cell division and differentiation. Synthetic herbicides used in the agriculture typically work in a similar fashion. As the plant kingdom is very diverse and thousands of species from this group produce vast array of chemical compounds possessing these properties, it might be possible to manufacture herbicides based on the allelochemicals naturally occurring in various plants, which could be more environmentally friendly than their synthetic counterparts. Additionally, most of the allelochemicals present in the plans are safe for humans and are typically not toxic for the soil or water^8^. In the agricultural and horticultural production, there is a growing interest in the use and production of seed meals, possessing before-mentioned allelochemicals^9,10^. However, their effect on the yield is not well understood. Additionally, seed meals, up to this day, have not been used in the research about quality of the wheat grain as a substrate for malt production.

## Results and discussion

### Technological parameters of the wheat malts

Analysis of the technological parameters of the malt was based on the basis of Congress mashing. Congress mashing is a typical method which is used in hundreds of breweries and malthouses to analyse performance of the malt in the brewhouse^1,11,12^. Results of these analyses are shown in Table 1.

**Table 1:** Technological properties of winter wheat malts.

Sample^a^	Saccharification time (min)	Filtration time (min)	Wort volume (cm^3^)	Wort extract (% w/w)	Wort pH	Wort viscosity (mPa s^−1^)
C	50	80 ± b	320 ± 5 b	8.57 ± 0.01 f	5.98 ± 0.01 ab	2.159 ± 0.002 a
HC	30	120 a	270 ± 5 c	8.55 ± 0.01 g	6.02 ± 0.02 a	2.084 ± 0.003 c
SA	10	72 ± 2 c	325 ± 5 b	8.95 ± 0.02 c	5.92 ± 0.02 d	1.941 ± 0.004 d
LL	10	45 ± 2 e	325 ± 5 b	8.82 ± 0.03 e	5.98 ± 0.01 ab	1.919 ± 0.001 e
FE	10	47 ± 1 e	330 ± 5 ab	8.98 ± 0.02 b	5.90 ± 0.02 d	2.136 ± 0.004 b
PT	10	46 ± 1 e	330 ± 5 ab	8.85 ± 0.03 d	5.97 ± 0.01 bc	2.166 ± 0.009 a
OS	10	60 ± 2 d	325 ± 5 b	8.97 ± 0.03 b	5.97 ± 0.01 bc	1.904 ± 0.005 e
RS	10	43 ± 3 e	340 ± 5 a	9.03 ± 0.03 a	5.94 ± 0.01 cd	1.911 ± 0.002 e

^a^Data are shown as means ± standard deviation (n = 2 in case of saccharification time, filtration time, wort volume; n = 6 in case of wort pH and wort viscosity). Different letters indicate homogenous groups analysed with the Tukey test (α = 0.05). Abbreviations are as follows: *C* malt produced from wheat grains grown without addition of seed meals or herbicides, *HC* malt produced from wheat grains grown without addition of seed meals, cultivated with herbicide treatment; *FE* malt produced from wheat grains grown with addition of seed meal from *Fagopyrum esculentum* Moench., *SA* malt produced from wheat grains grown with addition of seed meal from *Sinapis alba* L., *PT* malt produced from wheat grains grown with addition of seed meal from *Phacelia tanacetifolia* Benth., *LL* malt produced from wheat grains grown with addition of seed meal from *Lupinus luteus* L., *RS* malt produced from wheat grains grown with addition of seed meal from *Raphanus sativus* L. var *oleiformis* Pers., *OS* malt produced from wheat grains grown with addition of seed meal from *Ornithopus sativus* Brot.

One of the most important differences between analysed wheat malts is the saccharification time. Saccharification time of base malts should not be greater than 20 min, because it typically results in poor brewhouse efficiency and increases the costs of beverage production. All malts produced from wheat grains which were grown with addition of seed meals were characterised with excellent saccharification time, typical for well-modified Pilsner malt. Saccharification time of sample HC and C was significantly worse, equal to 30 and 50 min, which is higher than typical value acquired by darker pale barley malts, such as Vienna or Munich malt. Another advantage of using malts produced from wheat grains grown with the addition of seed meals can be seen in the filtration time of the worts produced in the Congress mashing procedure. Filtration time of the wort is an important parameter in the brewing industry, especially in large, industrial breweries, because it is one of the factors influencing the amount of brews which can be performed each day^1^. Additionally, worts produced from wheat malts or with addition of wheat malts are typically characterised by fundamentally longer filtration time than worts produced only from barley malts. Therefore, production of wheat malts characterised with so-called ‘normal’ filtration time could decrease production time of wheat malts. Complete filtration during the Congress mashing procedure should conclude up to 60 min to be called ‘normal’, while the filtration time of the worts which takes from up to 61 to 120 min is called ‘slow’. If the worts do not filter during 120 min, filtration is stopped and this result indicates an important flaw in the produced malt^10^. Sample HC acquired the worst result in the analysis of the filtration time (120 min) and had not filtered fully. Worts, which have acquired ‘slow’ filtration time, were C and SA. Samples RS, LL, PT and FE filtered in similar, normal time (43–47 min), while sample OS filtered slower, in 60 min. These results show an additional advantage of using seed meals for the production of wheat grains intended for malt production. Next Congress wort parameter is the volume of the acquired malt. The goal of the maltster is to produce malt with high extractivity^4^. Extractivity is the amount of substances, which can be extracted from the ground malt during the mashing procedure. Two parameters analysed during the Congress mashing regime show how much substances can be extracted from the malts and these parameters are wort volume and wort extract. Well modified malts with high enzymatic activity ought to yield a high volume of wort characterised with high extract^1^. Sample HC was characterised with lowest wort volume equal to 270 cm^3^ and samples FE, PT and RS acquired highest wort volume in the range of 330–340 cm^3^. Similar tendencies can be observed in the extract content of the produced worts. Samples HC and C were characterised with lowest extract content, ranging from 8.55 to 8.57% (w/w), while extract content of worts produced from samples SA, LL, FE, PT, OS and RS ranged from 8.82 to 9.03%, with the highest acquired by sample RS. Typical pH of the Congress worts produced from the barley malts should be equal to 5.6–5.9, but typical wheat malt Congress worts, according to Kunze^1^, are characterised by pH value close to 6. All the analysed samples acquired recommended pH values, with the lowest for FE (5.90) and highest for HC (6.02). Viscosity of the wort is another factor, which can prolong the process of beer production, by slowing the transfer of wort between various parts of the brewhouse. Worts produced from wheat malts ought to be characterised with viscosity lower than 1.550 mPa s^−1^^1^. All of the worts produced in this study acquired higher viscosity, with sample C and PT acquiring the highest (2.159 and 2.166 mPa s^−1^) and samples OS and RS the lowest (1.904 and 1.911 mPa s^−1^). All the results acquired by the Congress mash analysis suggest that using seed meals during the cultivation of wheat might not only be used only as a method for weed control, but also improve properties of the acquired malt and expand possibilities of utilising wheat grains by the farmers.

### Volatile compounds present in the wheat malts

Gas chromatography and mass spectrometry allowed for identification and quantification of 16 volatile components, shown in Table 2. The largest group of these components were aldehydes (9 compounds), while second and third groups constituted of alcohols (4 compounds) and esters (3 compounds). However, it is important to note that not all the wheat malts contained all of the identified volatiles. Sample C contained only 11 compounds, sample PT 12, sample HC, SA and LL 13, and sample OS 14. Sample RS was the only malt, in which all of the 16 compounds have been detected. Differences were not only in the number of compounds detected, but also in the overall quantity of volatiles present in the malts. The malts were characterised with concentration of volatiles ranging from 26.5 to 108.61 ppb. Highest concentration of volatile compounds was detected in sample LL, while lowest in sample PT.

**Table 2:** Volatile compounds in winter wheat malts.

		Chemical family	Aroma^a^	C^b^ (ppb)	HC (ppb)	SA (ppb)	LL (ppb)	FE (ppb)	PT (ppb)	OS (ppb)	RS (ppb)
1	1-Octen-3-ol	Alcohols	Mushroom, earthy	1.46 ± 0.12 d	n.d	n.d	5.42 ± 0.04 a	3.86 ± 0.09 b	n.d	2.18 ± 0.06 c	0.94 ± 0.06 e
2	Octanal	Aldehydes	Aldehydic, waxy	n.d	5.13 ± 0.18 b	7.66 ± 0.19 a	1.72 ± 0.14 c	0.53 ± 0.14 e	1.11 ± 0.12 d	n.d	0.41 ± 0.05 e
3	1-Hexanol, 2-ethyl-	Alcohols	Citrusy	n.d	n.d	n.d	n.d	n.d	n.d	0.81 ± 0.15 a	0.57 ± 0.09 b
4	2-Octenal, (E)-	Aldehydes	Cucumber-like	1.70 ± 0.24 a	0.23 ± 0.04 d	0.34 ± 0.04 c	n.d	0.35 ± 0.08 c	n.d	n.d	0.75 ± 0.14 b
5	1-Octanol	Alcohols	Waxy, orange-like	n.d	n.d	n.d	n.d	n.d	n.d	1.63 ± 0.30 a	2.05 ± 0.36 a
6	4-Nonenal, (E)-	Aldehydes	Fruity,	6.31 ± 0.15 b	2.32 ± 0.22 de	2.07 ± 0.20 e	7.59 ± 0.65 a	5.82 ± 0.14 c	1.58 ± 0.34 f.	2.73 ± 0.30 d	2.55 ± 0.37 d
7	Nonanal	Aldehydes	Waxy, rose-like, orange-like	5.53 ± 1.14 fg	9.35 ± 1.38 cd	11.67 ± 1.30 b	14.88 ± 1.07 a	7.89 ± 1.87 de	5.22 ± 0.71 g	4.11 ± 0.52 h	6.93 ± 1.12 ef
8	2,6-Nonadienal, (E,Z)-	Aldehydes	Cucumber-like	2.31 ± 0.50 b	1.68 ± 0.34 cd	0.78 ± 0.09 e	3.63 ± 0.37 a	1.68 ± 0.14 c	0.48 ± 0.09 f.	0.63 ± 0.08 e	1.36 ± 0.28 d
9	2-Nonenal, (E)-	Aldehydes	Cucumber-like, citrusy	37.56 ± 2.74 a	16.36 ± 2.53 c	10.50 ± 1.17 d	36.97 ± 2.68 a	27.72 ± 1.66 b	6.11 ± 1.24 e	11.36 ± 1.42 d	15.44 ± 2.74 c
10	1-Nonanol	Alcohols	Floral, rose-like	n.d	1.92 ± 0.32 b	2.35 ± 0.47 a	2.22 ± 0.13 a	1.16 ± 0.24 c	0.82 ± 0.11 d	0.72 ± 0.18 de	0.59 ± 0.09 e
11	Octanoic acid, ethyl ester	Esters	Fruity,	1.72 ± 0.29 a	0.84 ± 0.14 d	0.97 ± 0.17 cd	1.41 ± 0.19 b	1.01 ± 0.26 c	0.34 ± 0.07 f.	0.54 ± 0.12 e	0.20 ± 0.07 g
12	Decanal	Aldehydes	Waxy, citrusy, floral	8.53 ± 2.27 d	24.26 ± 3.61 b	43.08 ± 4.65 a	24.72 ± 1.93 b	15.86 ± 3.79 c	8.20 ± 1.66 d	4.22 ± 0.54 e	8.26 ± 1.23 d
13	Acetic acid, 2-phenylethyl ester	Esters	Floral, honey-like	2.09 ± 0.35 b	1.23 ± 0.17 c	1.04 ± 0.08 d	2.61 ± 0.46 a	1.14 ± 0.28 cd	0.72 ± 0.14 f.	0.88 ± 0.10 e	0.93 ± 0.16 e
14	Undecanal	Aldehydes	Soapy, aldehydic	0.88 ± 0.26 c	1.05 ± 0.13 b	1.71 ± 0.23 a	1.74 ± 0.13 a	1.07 ± 0.23 b	0.66 ± 0.13 d	0.44 ± 0.06 e	0.61 ± 0.07 d
15	Decanoic acid, ethyl ester	Esters	Fruity, apple-like	0.96 ± 0.18 b	0.80 ± 0.15 c	0.51 ± 0.10 e	1.66 ± 0.23 a	0.61 ± 0.13 d	0.31 ± 0.05 g	0.41 ± 0.06 f.	0.62 ± 0.14 d
16	Dodecanal	Aldehydes	Soapy, aldehydic,	n.d	0.51 ± 0.12 d	0.64 ± 0.16 c	1.42 ± 0.17 a	0.88 ± 0.19 b	0.50 ± 0.06 d	0.29 ± 0.08 e	0.48 ± 0.14 d

^a^As decribed by Gao et al., McGorrin and Czerny et al.^13–15^.

^b^Data are shown as means ± standard deviation (n = 3). Different letters indicate homogenous groups analysed with the Tukey test (α = 0.05). *N.d* not detected. Abbreviations are as follows: *C* malt produced from wheat grains grown without addition of seed meals or herbicides, *HC* malt produced from wheat grains grown without addition of seed meals, cultivated with herbicide treatment, *FE* malt produced from wheat grains grown with addition of seed meal from *Fagopyrum esculentum* Moench., *SA* malt produced from wheat grains grown with addition of seed meal from *Sinapis alba* L., *PT* malt produced from wheat grains grown with addition of seed meal from *Phacelia tanacetifolia* Benth., *LL* malt produced from wheat grains grown with addition of seed meal from *Lupinus luteus* L., *RS* malt produced from wheat grains grown with addition of seed meal from *Raphanus sativus* L. var *oleiformis* Pers., *OS* malt produced from wheat grains grown with addition of seed meal from *Ornithopus sativus* Brot.

It is essential to indicate that high concentration of volatile compounds in the base malt (i.e. pale malt) samples is not preferred by the maltster and a brewer, because most of the volatile compounds present in pale malts do not have aroma typical for beer^16,17^. Most of the volatiles present in the finished product is produced by yeast and malt, used in the process of brewing, brings to the wort only components needed for the creation of expected volatiles, such as sugars and amino acids, which can then be used by the yeast for the production of expected volatiles, such as esters (for example, ethyl hexanoate or ethyl octanoate) and alcohols (such as 1-propanol) which are present in beer in far greater quantity than in grain (even in the range of hundreds of ppm, not ppb, as seen in the malt samples)^18^. Unfortunately, some volatile components present in the malt samples can have a significantly debilitating effect on the aroma of fermented beverages, which is why it is better to produce pale malt with the lowest possible concentration of particular metabolites^19^. One of the most important components of such type, which was found in all the analysed malt samples, is (E)-2-nonenal (trans-2-nonenal). It is a compound with a very low taste threshold and is responsible for so-called ‘cardboard off-flavour’ in fermented beverages^20,21^. Higher concentration of this component might be the result of oxidation of lipids and fatty acids, various Strecker degradations, aldol condensation of short chain aldehydes or secondary oxidation of long chain aldehydes, but data gathered in this study cannot precisely pin-point exact mechanism^19^. It can be seen that only some of the malts produced from the grain grown with the use of seed meals (SA, PT, RS, OS) had (E)-2-nonenal concentration in the range similar or lower to the sample HC, grown without seed meal addition. It is important to note, that the (E)-2-nonenal concentration in these samples was in the range accepted for the typical barley malts equal to 1.9–61.7 ppb^22^. Additional groups of interesting compounds, which were detected in the malt samples, were volatile components typically produced by the various microorganisms, such as yeast, bacteria or fungi^18,23–25^. These components, detected in the malt samples, were octanoic acid, ethyl ester; acetic acid, 2-phenylethyl ester and decanoic acid, ethyl ester. Similarly to the concentration of (E)-2-nonenal, samples SA, PT, RS and OS were characterised with lower concentration of these compounds than the sample C. Additionally, concentration of these components in samples PT, RS and OS was lower than the concentration in the HC sample, which might show that there is potential in using these seed meals not only as a way of herbicide-control, but possibly these preparations can have some anti-microbial properties as well. However, more research specifically aimed to determine these parameters is needed to confirm these hypotheses. Nevertheless, conducted study shows that using seed meals during winter wheat sowing has an impact on the volatile composition of the wheat malts produced from seeds of these plants, albeit precise mechanisms of these changes have to be analysed in the future.

## Materials and methods

### Plant material

Plant materials used in this study were certified grains of winter wheat cv ‘Agil’ acquired from Syngenta company (Basel, Switzerland). The field experiment (split-plot design, with three plots as replication) was carried out at Wrocław University of Environmental and Life Science’s Research and Training Station in Swojczyce in Poland (51° 07′ N 17° 08′ E) in the vegetative season 2020/2021. The experimental factor was the type of seed meal of qualified seeds of selected crops (*Fagophyrum esculentum*, *Sinapis alba*, *Phacelia tanacetifolia*, *Lupinus luteus*, *Raphanus sativus*, *Ornithopus sativus*). The preparation of seed meals and their characterization was described in an article by Pytlarz and Gala-Czekaj^7^. The next day after the sowing wheat the seed meals were sown in the plots (dose 100 g m^−2^) and mixed with a hand rake. The control and herbicide control fields did not contain any addition of seed meals. The herbicide control treatment was sprayed on the two tillers detectable of unfolded-stage (BBCH 22) winter wheat. The dose of propoxycarbazone-sodium was 56 g ha^−1^ per 200 dm^3^ of water (selective herbicide Attribut 70SG). It is classified by HRAC as an Herbicide MoA Group 2. The active substance has a systemic effect. The plots were sprayed with fungicide (Artea 330 EC in dose of propiconazole 125 g ha^−1^ and cyproconazole 40 g ha^−1^) and insecticide (Decis Mega 50 EW with a dose of deltamethrin equal to 0.006 g ha^−1^) as the risk emerged. Due to high phosphorus and potassium contents in the soil (128 mg of phosphorus and 125 mg of potassium per kg of soil), pre-sowing fertilisation with these macronutrients was not applied. Nitrogen fertilisation was applied in the dosage of 120 kg of nitrogen per hectare in three separate doses: 120 kg N ha^−1^ (using 34% ammonium sulphate) before sowing; 30 kg N ha^−1^ (using 46% urea) at the start of vegetation (BBCH 22–23) and 30 kg N ha^−1^ (using 34% ammonium sulphate) at the time of stem elongation (BBCH 37–39). All methods were carried out in accordance with relevant institutional, international and national guidelines and legislation.

### Reagents and standards

Reagents and standards used in this study was cyclohexane (99.9%) and 2-undecanone (99.9%) (Sigma Aldrich, Saint Louis, MO, USA) which were used as an internal standard for the gas chromatography (as a solution of 1 mg of 2-undecanone per 1 dm^3^ of cyclohexane).

### Malting technology

#### Grain sifting and analyses of grain basic parameters

Prior to malting, the grains were cleaned from impurities and sorted through sifting with the use of Laboratory Sorting Machine Sortimat (Pfeuffer GMBH, Kitzingen, Germany). Grains with any of the dimensions lower than 2.5 mm were weighed and discarded. Moisture content was analysed with the use of the Infratec 1241 Grain Analyzer (Foss, Hilleroed, Denmark).

#### Steeping and germination

Seventy gram portions of wheat grain were weighed and transferred into perforated stainless steel malting containers. Combined weights of the container and grain (from now on referenced as a ‘malting kit’) were measured. Changes in the moisture content of the grain during the steeping process were assessed by the changing weight of the malting kits, with the assumption that the increase in weight is equal to the weight of the water absorbed by the grains. Before steeping, grains were disinfected by submerging malting kits in sodium hypochlorite solution (1.5% v/v) for 10 min, after which they have been rinsed in sterile, distilled water (15 °C). Steeping was executed by submerging malting kits in tap water for 5 h (at temperature of 15 °C); removing containers from the water and storing them in a humid air in a KK 240 Smart Pro germination chamber (Pol-Eko Aparatura, Wodzisław Śląski, Poland) (90% relative humidity, 15 °C) for 19 h; submerging containers in fresh tap water for 5 h (15 °C) and performing last air rest of grain steeping by storing the containers in the germination chamber (90% relative humidity, 15 °C) for another 19 h. After 48 h of steeping sequence, grains acquired moisture content equal to 45%.

Grain was kept in the refrigerated germination chamber (at 15 °C and 95% relative humidity) for 120 h. Every 24 h, grains were mixed manually to prevent root entanglement and were sprayed with distilled, sterile water to eliminate water deficiencies and maintain constant humidity throughout germination.

Stainless steel containers, before loading wheat grains into them, were disinfected by storing them in the oven set at 200 °C for 3 h.

#### Kilning and grinding

Kilning of the germinated grains was performed immediately after germination. Malting kits were loaded into a UF110 Plus dryer (Memmert GmbH+Co, Schwabach, Germany) and kilned with following conditions: 50 °C (17 h and 50 min), ramp up to 65 °C (10 min), 65 °C (2 h and 50 min), ramp up to 82 °C (10 min), 82 °C (2 h). After kilning, malt was transferred into tightly closed containers, which prevented moisture absorption during the cooling period. After the temperature of malt dropped to 20 °C, rootlets of the malt were manually removed and malt was ground on the Bühler Miag disc mill DLFU (Bühler, Uzwil, Switzerland), according to the Analytica EBC 4.5.1 method^1,11^.

Malting procedure allowed for production of subsequent malts:

- malt produced from wheat grains grown without addition of seed meals or herbicides (C)
- malt produced from wheat grains grown without addition of seed meals, cultivated with herbicide treatment (HC)
- malt produced from wheat grains grown with addition of seed meal from *Fagopyrum esculentum* Moench. (FE)
- malt produced from wheat grains grown with addition of seed meal from *Sinapis alba* L. (SA)
- malt produced from wheat grains grown with addition of seed meal from *Phacelia tanacetifolia* Benth. (PT)
- wheat grains grown with addition of seed meal from *Lupinus luteus* L. (LL)
- malt produced from wheat grains grown with addition of seed meal from *Raphanus sativus* L. var *oleiformis* Pers. (RS)
- malt produced from wheat grains grown with addition of seed meal from *Ornithopus sativus* Brot. (OS)

### Analysis of the technological parameters of wheat malts

Congress worts were produced in the automated mashing machine (LB Electronic, Berching, Germany) according to the Analytica EBC 4.5.1 method^11^. Wort, after filtration, was collected for analyses. Worts were prepared in duplicate.

#### Wort saccharification time

Saccharification time was assessed by the Analytica EBC 4.5.1 method^11^. At duration of 10 min after adding water at a temperature of 70 °C to the mashes, measurement of saccharification time was started and iodine tests were performed in 5 min intervals. The last tests were performed in the interval of 55–60 min of mashing at a temperature of 70 °C.

#### Wort pH

Wort pH was assessed with a pH-meter (MP220, Mettler Toledo, Columbus, OH, USA) in worts collected after the filtration process (at 20 °C). Measurement was performed in triplicate for each wort sample (total of six measurements each type of malt).

#### Wort extract content

The extract content of the worts was assessed with the use of densimeter (DMA 35, Anton Paar, Graz, Austria) in the worts with temperature adjusted to 20 °C. Measurements were performed in triplicate for each wort sample (six measurements per type of malt).

#### Wort filtration time

Analysis of the wort filtration time was performed by the Analytica EBC 4.5.1. method^11^, up to 120 min of filtration time, which resulted in two readings per one type of malt.

#### Wort volume

Wort volume was recorded from the scale of the graduated cylinder after 120 min of the filtration process, which resulted in two readings per one type of malt.

#### Wort viscosity

Wort viscosity test was performed by the Analytica EBC 8.4. method^26^ using KF10 Viscometer (Messtechnik GmbH, Ottendorf-Okrilla, Germany). Three readings were performed for each of the congress worts, resulting in six results for each malt type.

### Analysis of the volatile compounds in wheat malts

#### Adsorption of volatile compounds to the solid-phase microextraction fiber

To perform chromatographic analysis of volatiles present in the malt samples, the volatiles had to be adsorbed on the solid phase microextraction fiber (SPME)^13^. Two grams of the ground malt were added to the 20 cm^3^ headspace vial, followed by the addition of 0.5 g sodium chloride and 4 cm^3^ of distilled water. Twenty nanograms of internal standard (2-undecanone) was added to the vial, which was then closed with a magnetic screw-top cap with a septum. SPME holder needle, equipped DVB/CAR/PDMS fiber (50/30 µm) (Supelco, Bellefonte, PA, USA) was used to pierce the septum. Vial was positioned on the heatplate set at 50 °C. After 5 min of temperature equilibration, the fiber was extended from the holder needle, to allow adsorption of the volatiles on the fiber surface for 50 min. After adsorption of the volatiles, fiber was retracted into the holder. Each of the malt samples was analysed in triplicate.

#### Gas chromatography and mass spectrometry

Gas chromatography and mass spectrometry of the volatiles was performed on the GC-2010 Plus chromatograph coupled with GCMS-QP2010 SE mass spectrometer (Shimadzu, Kyoto, Japan) equipped with ZB-5 column (Phenomenex, Torrance, CA, USA) (30 m length × 0.25 mm internal diameter × 0.25 µm film thickness). Injection port temperature was held at 195 °C. Analyses were carried out with the use of helium as a carrier gas with a flow rate of 1.78 cm^3^ min^−1^ and a starting pressure set at 100 kPa. Following program was used for the oven temperature: 40 °C at the beginning; hold for 1 min, ramp up at a rate of 8 °C min^−1^ to 195 °C; hold for 5 min. Ion source temperature was maintained at 250 °C, while interface temperature was at 195 °C. Scanning was carried out in the 35–350 m/z range using 70 mV electron ionisation with the event time equal to 0.3 s (scan speed equal to 1111). Volatile compounds separated from the grains and malts were identified by mass spectral analysis, comparing retention indices with Kovats standards and with NIST17 chemical standard libraries.

### Data analysis

The results of the analysis of technological parameters of malts and concentration of volatiles were statistically analysed in the SPSS Statistics 26 program from IBM (Armonk NY USA) using one-way ANOVA with α = 0.05 using the Tukey test. Chromatographic peaks were integrated with the use of Shimadzu PostRun Analysis program (Shimadzu, Kyoto, Japan).

## Conclusions

Production of wheat malt from the grains of winter wheat plants grown with the addition of seed meals into soil is possible and seems to have various advantages. Malts produced from these grains were characterised with improved technological properties, such as faster saccharification time, shorter filtration time, higher wort volume and higher wort extract. However, seed meals from *Fagopyrum esculentum* Moench. and *Lupinus luteus* L. had an debilitating effect on the composition of volatiles present in the winter wheat malts (compared to the herbicidal control), such as higher concentration of (E)-2-nonenal, 2,6-(E,Z)-nonadienal or nonanal. These results show that all tested seed meals could be used to improve technological properties of wheat malts, albeit some caution ought to be applied during selecting proper seed meal, if the farmer and maltster would like to produce malt with low quantity of particular volatile metabolites.

## Data Availability

The datasets used and analysed during the current study are available from the corresponding author on reasonable request.
